# The effect of macroscopic herd inputs on individual investment behaviour

**DOI:** 10.1038/s41598-024-53946-9

**Published:** 2024-02-08

**Authors:** Kristian Roed Nielsen, Micha Kaiser, Fumiko Kano Glückstad

**Affiliations:** 1 Department of Management, Society and Communication, Copenhagen Business School, Dalgas Have 15, 2000 Frederiksberg, Denmark; 2 Cambridge Judge Business School, El-Erian Institute of Behavioural Economics and Policy, University of Cambridge, Trumpington Street, Cambridge, CB2 1AG UK

**Keywords:** Human behaviour, Behavioural ecology, Social behaviour

## Abstract

Decisions are rarely made in isolation and the role of others’ decisions in guiding our own has been observed in a diversity of contexts. This influence is often argued to result from an information cascade, where decisions in a sequential setting are influenced by the early decisions of others. However, the degree to which individuals modify behaviour through the integration of social information (i.e., other people’s decisions) varies considerably. While significant literature has been dedicated to understanding individual determinants for this variation, we propose that we should not ignore the aggregate characteristics of the herd itself. Specifically, we examine whether the scale and longevity of the herd itself at the time when an individual decides, defined as macroscopic herd inputs, influence whether individuals integrate social information. By employing data from a social trading platform, we find that macroscopic herd inputs exert a strong influence on individual investment decisions, showing that the influence of others’ behaviour on our own is in part dependent on the nature of the herd itself.

## Introduction

Decisions are rarely made in isolation and thus the role of others decisions in guiding our own plays a significant role especially in conditions of uncertainty^1^. We look to others when choosing a restaurant^2^, picking what to read^3^, voting^4^, and making financial decisions^5,6^. Given the many situations, where we make decisions in the presence of others doing the same, understanding the mechanics of these influences on our behaviour seems as pertinent as ever. Especially as limited time and imperfect decision-making processes mean that herding can result both in positive and negative decision outcomes^7^. We define herding as the intentional alignment of the thoughts and behaviour of individuals to those of a group without centralized co-ordination^8^.

How individuals derive information from the actions of others is often conceived to emerge as a result of an information cascade, where decisions in a sequential setting are strongly influenced by the early decisions of others^9,10^. In extreme circumstances, causing later-deciding individuals to abandon their own private information and instead adopt a behaviour based on what everyone else is doing^7^. At this point, individual decisions become uninformative to others and subsequent individuals drawing inference from the history of past decisions results in an information cascade. Resulting critically in a situation where the optimal action of the individual becomes to adopt a specific behaviour dictated not by private information, but the past (un)informed decisions of the former individuals^9,11^. From this perspective, deriving decisions from the actions of others thus becomes fraught with problems as decisions can equally result in both correct and incorrect cascades. Furthermore, because individuals can only observe decisions and not the information that it was based on, it becomes impossible for subsequent investors to differentiate when social information is informative or hazardous^12^.

However, the degree to which individuals incorporate social information into their own varies significantly for example due to different levels of personal information, different decision thresholds, how individuals' mentalize the actions of others, and conformity preferences^7,8, 13^. In addition, to these factors, we propose that the scale and longevity of the herd at the time when an individual decides, defined as macroscopic herd inputs, has a marked influence on whether an individual modifies their behaviour based on the actions of others. The introduction of the concept of macroscopic herd inputs is inspired by the literature on collective animal behaviour^14,15^ and in particular the work by Raafat et al.^8^ and Sosna et al.^1^ on global or macroscopic patterns of interaction in herds. This research examines how aggregate-level herd behaviour, as opposed to individual-to-individual cascades, influences individual responses. This form of research drawing parallels to notions that herds may even be independently “wise”^16^ and that individuals in groups may thus “gain access to higher-order collective computational capabilities […] from widely distributed sources”^14^. These macroscopic herd inputs providing in the moment social feedback that may act to change the individuals’ subjective level of evidence required for them to make or alter a decision^7^. The evolutionary value of macroscopic herd inputs is that it provides a source of alternative information from a wider distributed source of individuals based on their actions^14^. Thus, our paper seeks to examine *whether and how macroscopic herd inputs influence individual decision-making in an investment context*.

Our study is interested in empirically examining when sequential decisions-making may or may not be influenced by macroscopic herd inputs, which is done by examining how larger herd activity may act to inform an individual on the usefulness of others actions and in turn increase the likelihood that they adapt their behaviour in accordance. Therefore, based on these observations we propose that the given intensity and persistency of a herd—the level of activity and the amount of time that the herd has been active—provides individuals with a significant source of social information.

In order to examine our research question, we draw on a lending-based crowdfunding context as the ‘scopic regime’ of this type of social trading platform provides an ideal context for studying herding^17^. Scopic regimes referring to investment contexts which “designates a state of permanent reciprocal observation and scrutiny”^6^. In practice individual investors on these platforms can observe not only campaign-specific investment details, but also the details of all the investments made by others, including the timing and the amounts of those investments^18,19^. This provides an ideal context to observe herds as decisions are sequential with subsequent actors observing decisions of previous actors and there is a limited action space^12^. By observing how herd and non-herd contexts influence individual decision-making we demonstrate that modelling macroscopic herd inputs makes it possible to estimate when and to what degree individuals employ social information from the herd to guide their own behaviour. Specifically, we show that the scale or frequency of investments (intensity) and the longevity or duration of herd activity (persistency) of other actors at the time when an individual makes an investment exerts a strong influence on individual investment decisions. Thus, showing that the degree to which individuals adopt behaviour like that of those who came before them is influenced significantly by the social information that macroscopic herd inputs provide individuals. Indeed, we find that increasing herd activity, contrary to what we might expect from an information cascade, results in individuals investing significantly less than what they do on average.

## Results

**Figure 1:**
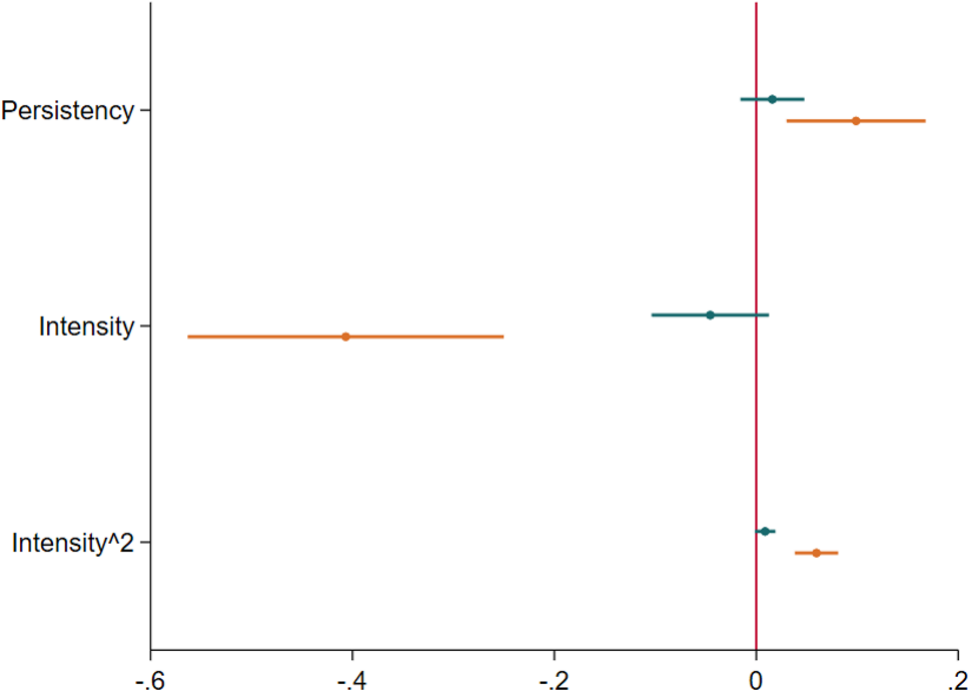
Marginal effects of intensity and persistency on average investment. The graph shows the coefficients of two separate OLS regression models of individual investment on a log-transformed scale for intensity and persistency and several controls following the approach of Li et al.’s^20^. Model 1 for “No Herding” (in blue) is estimated based only on investments made when herding behaviour was not present, while Model 2 for “Herding” (in red) uses only observations where herding behaviour occurred. Both models include the same covariates, with standard errors clustered at the campaign level. To capture the non-linear form of intensity, we included second-order polynomial terms. The dots indicate the effect size, with values indicated on the x-axis, while the bars show the estimated 95% confidence intervals.

**Figure 2:**
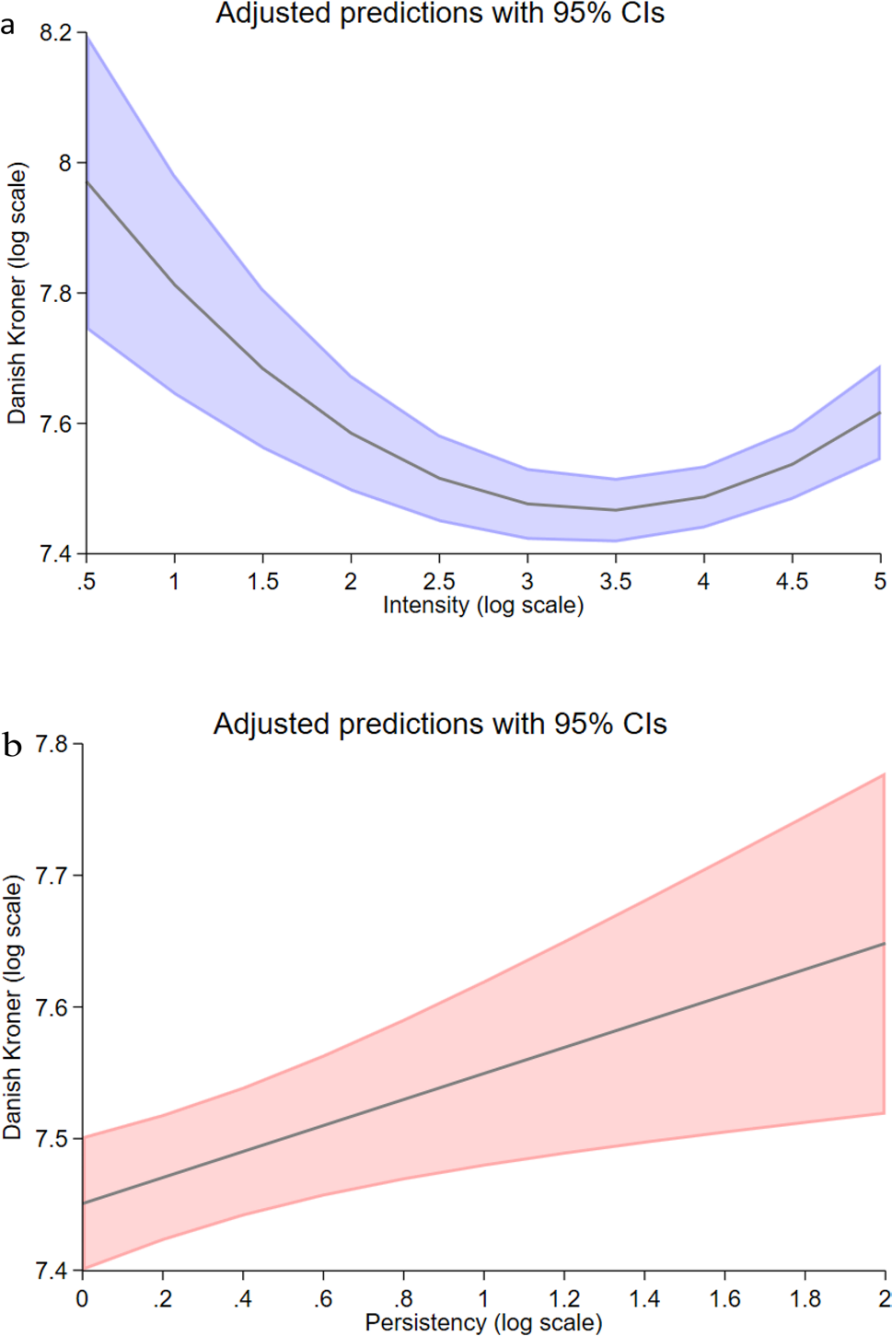
The influence of intensity and persistency on individual investment behaviour. Linear predictions of an individual’s average investment are based on intensity (**a**) and persistency (**b**) (in log form), while holding the covariates constant and setting them to their respective sample means. The shaded areas indicate 95% confidence intervals. Predictions are calculated based on model specification 2 (see Table 2 method), which employed only observations when herding is present.

We observe that macroscopic herd inputs significantly influence individual investment behaviour, but also that these effects are largely herd dependent. Figures 1 and 2 provide a visualization of the respective effects of intensity and persistency on investor behaviour measured in DKK invested (in log). Figure 1 provides an overview of the respective effects of intensity and persistency in both herding and non-herding contexts as compared to the average baseline investment of individuals (vertical red line). From this figure (and Table 2) we observe that while intensity and persistency have a large effect in our model focused exclusively on observations during herding, we also find that persistency and intensity only have minimal influence on investor behaviour in non-herding contexts. This serves to bolster the argument that the operationalization of macroscopic herd inputs as intensity and persistency is valuable and empirically useful to studying the influence of herds on behaviour. Figure 2 outlines the respective effects of intensity (intensity^2) and persistency on behaviour in herding context only.

In examining the influence of herd intensity (panel a) on individual investments, it is evident that the positive feedback loop we would expect from increasing herd activity, i.e. more investor activity, is not empirically observable. Notably, the analysis reveals that increasing macroscopic herd intensity overall significantly reduces the average investments from the respective investor, however, this relationship is not linear. Rather a u-shaped relationship is apparent where increasing intensity initially is associated with a fall in average investments, but ultimately kinks at higher levels of intensity resulting in a positive relationship. This suggests that the relationship between macroscopic intensity and investor behaviour has diverging positive and negative effects at the individual level. This dynamic nature reveals that the likelihood that individuals will modify their behaviour based on the actions of others is neither a positive nor negative feedback loop but are subject to dynamic variations in day-to-day herd intensity. This day-to-day variation in herd intensity and in turn its significant effect on investor behaviour may help explain the significant variation we observe in how and in what degree individuals are influenced by others.

If we turn our attention to herd persistency (panel b), we conversely find that herd persistency—the longevity of herd activity—significantly increases average individual investment. Thus, while investors appear to react with initial skepticism towards increasing investor activity, the longevity of that activity is treated as a positive sign. Investors appear to differentiate between macroscopic herd inputs, whereas increases in herd intensity is treated with skepticism persistent herd activity translates into significantly increasing average investments. The variability in the effect of information cascades on individuals may therefore be linked not only to individual characteristics, but also variations in how the herd is expressed on aggregate. Our results thus both illustrate the significant impact that macroscopic herd inputs have on investor behaviour, but also the variability of how two different forms of macroscopic herd inputs are treated differently by the same investor.

## Discussion

The effect of herding on human behaviour is often cast in the light of an information cascade that emerges in contexts where people make sequential decisions with a limited action space. These cascades ultimately being error prone as individuals’ are conceived to forgo private information and instead adopt the behaviour of those who came before^10,21^. We proposed that while individual-to-individual cascades in many contexts do influence behaviour, we were interested in examining whether macroscopic herd inputs also influences behaviour as inspired by the literature on collective animal behaviour^14,15^. We find that macroscopic herd inputs have a significant influence on the extent to which individuals draw on the actions of others as a source of information. More specifically, we observe that macroscopic inputs in the form of the intensity and persistency of the herd at the time when an individual decides strongly influences the manner and the degree to which individuals use the actions of others as a source of information to modify their own behaviour. Indeed, we find that macroscopic herd inputs proved significantly better at modelling herd effects on individual behaviour than the mere absence or presence of a herd. We thus find compelling empirical evidence that global^8^ or macroscopic^1^ herd inputs do provide individuals with information that influences whether or not they use the actions of others as a source of information to modify their own behaviour and the extent to which they do so. Our study thus provides empirical insights both into herding behaviour among humans generally and the specific mechanics of how herds may provide individuals with social information^14^. Our findings further provide useful insights into the potential variability of the effect of herds on individual behaviour. We thus add to the initial findings of Tump et al.^7^ in showing that the quality of information cascades is influenced not only by person-specific attributes, but also time specific macroscopic herd inputs that significantly shape the degree to which an individual employs the actions of others to guide their decisions. Critically we empirically show that while individual-to-individual cascades of information certainly play their role in guiding the integration of social information, one should not ignore larger herd inputs, or one runs the risk of deconstructing a herd to simply being a matter of individual-to-individual interaction. Given the complexities and risks involved in making investment decisions, we propose our findings will be relevant to other areas of online collective decision-making in which individuals make decisions in a sequential manner in conditions of uncertainty.

Considering the importance that herding plays in many day-to-day practices quantifying and understanding its influence on behaviour are as important as ever. Especially considering the contested research surrounding herding, both in terms of its irrational^22^ or rational nature^23,24^, but also regarding the proposed wisdom related to following herds. Where from one perspective the convergence of behaviour based on the behaviour of others is seen as idiosyncratic, fragile and error-prone^9,10^ others argue that this form of observational or social learning have evolutionary value that provide individuals with information from widely distributed sources^14,25^. Having quantified and measured the significant effect that macroscopic herd inputs have on investor behaviour we believe our findings could provide research and practice with an alternative means to engage in the debate on the nature of herding. By seeing herding not only as a series of individual-to-individual interactions, but also as a larger aggregate phenomenon we may better understand when herds act as sources of useful alternative information or as a fragile cascade of ill-informed decisions.

### Limitations and avenues for future research

Simons et al.^26^ note that all research is bound by its specific sample, materials, procedures, and historical or temporal setting that give rise for opportunities for further investigation. The Danish context in which we derive our observations significantly influences the degree to which our findings can be generalized beyond these confines. For example, the high level of social trust in Denmark may influence the degree to which individuals are willing to trust the actions of others as a source of reliable information^27^. Cross-cultural comparison of our findings with countries exhibiting lower levels of social trust could consequently provide insights into whether macroscopic herding is dependent on the institutional context. Secondly, while our data provides us with in-depth longitudinal and granular level of data on actual funder behaviour, we lack the counterfactual conditions that would allow us to establish clear causality on the observed relationships^28,29^. Future research could thus seek to expand on our findings in an experimental setting and observe how manipulations in herd persistency and intensity influence investor behaviour, but also observe how variations in individual characteristics changes the process of assimilating social information. Thirdly, our data is limited as we lack a good risk classification measure assigned to the respective projects. Considering that Gemayel and Preda^6^ have shown that risk-seeking investors are less prone to herding, insights into investor risk preferences could have helped both explain some of the heterogeneity in our results and potentially also have nuanced our observations as to who responds to herd inputs. Finally, our focus on online decision-making creates obvious limits to the degree to which our observations can be replicated. For example, the visceral and physical nature of in-person herds contextually differ greatly from behaviour in online circumstances.

## Material and methods

### Dataset and empirical context

The emergence of crowdfunding as an alternative means of financing to mainstream sources of innovation finance has received significant academic interest not least given its exceptional growth and its touted democratizing facets^30,31^. The crowdfunding process itself “characterized by the successful interaction between a facilitating organization (or platform), a variety of campaign founders who seek financial support for their ideas and ventures, and a large dispersed “crowd” of individuals (“crowdfunders”) who are enticed to invest, pledge, lend, or donate money”^32^. The process depending on the entrepreneurs’ ability to mobilize support from a broad community of strangers in order to mobilize resources and tap into crowd judgement^33,34^.

The reliance of crowdfunding on numerous small and transparent investments from a dispersed and heterogeneous collection of individuals^35^ also provides an excellent context for exploring the phenomenon of herding^17,20, 36^. The structure of crowdfunding platforms enables individuals to observe not only campaign-specific investment details but also the details of all the investments made by others, including the timing and the amounts of those investments^18,19^. This means that individuals can use this information about others’ investment behaviour in evaluating a campaign and in deciding whether and how much to invest. These signals are additionally important as there is no third party financial certification/endorsement in crowdfunding^17^. This form of ‘scopic regime’ where the platform “designates a state of permanent reciprocal observation and scrutiny”^6^ provides an ideal empirical context to study herding. Accordingly, we can calculate how macroscopic herd inputs influence behaviour by observing how the same individual adjusts their investment sums based on variations in the macroscopic herd inputs at the time when they make their investment.

The data is derived from a Danish lending-based crowdfunding platform Lendino.dk that accounts for a significant amount of the capital raised via crowdfunding in the Danish context^37^. The data was provided by the platform and is therefore considered to offer a comprehensive picture of all the campaigns hosted on their platform from 2014 up to and including April 2021. As our main outcome variable, we use the individual amount invested to a specific campaign as measured in Danish Kroner (DKK). The covariates used in the analysis are based on well-established empirical strategies commonly used in the literature (see for example Mollick 2014﻿^35^). These variables include both campaign and individual-level characteristics and the campaign year, month, and weekdays to account for any temporal effects that are independent of herd formation. Table 1 of the descriptives statistic provides an overview of the control variables.

**Table 1:** Descriptive statistics for all variables in the final dataset.

Variable	Mean/%	SD	Min	1st Quartile	Median	3rd Quartile	Max	N
Campaign specific controls								
Funding goal (Danish Kroner)	412,522	254,646	25,000	250,000	350,000	500,000	3,100,000	27,475
First herd event for campaign*								12,020
No	17.34							2084
Yes	82.66							9936
Campaign specific sector								27,475
Building and construction	2.09							574
Property	3.24							889
Finance	0.20							54
Trading	38.88							10,683
IT	2.77							760
Industry	8.73							2399
Agriculture, horticulture and forestry	2.68							735
Life science	0.55							150
Service industries	36.20							9947
Transport	2.18							600
Tourism	2.49							684
Funder specific controls								
Campaign year^+^								27,475
2014	0.6							164
2015	8.44							2319
2016	30.61							8409
2017	31.75							8724
2018	14.85							4079
2019	5.94							1623
2020	6.33							1738
2021	1.49							410
Campaign month^+^								27,475
January	10.47							2876
February	6.5							1787
March	7.41							2035
April	8.08							2219
May	6.34							1743
June	7.92							2177
July	7.49							2059
August	8.39							2305
September	9.24							2539
October	9.79							2691
November	40.52							2890
December	7.84							2154
Campaign weekday^+^								27,475
Monday	15.86							4357
Tuesday	18.94							5203
Wednesday	19.26							5293
Thursday	19.08							5241
Friday	19.32							5307
Saturday	4.23							1162
Sunday	3.32							912
Gender								27,475
Male	88.5							22,396
Female	11.5							2930
Birth Decade								25,326
1920	0.01							3
1930	0.48							121
1940	7.73							1985
1950	15.26							3865
1960	20.07							5082
1970	24.92							6310
1980	25.54							6468
1990	5.99							1516
2000	0.01							3
Funder experience: (number of previous investments)	27.42	32.60	1	6	16	36	275	27,475
Geographic distance between funder and campaign in kilometers	108.21	82.25	0	26.44	107.53	177.95	430.2	25,801
Number of days passed since the campaign started	4.69	15.36	0	0.02	0.166	3.31	358	27,475
Funder risk (average number of days passed since the campaign started)	4.69	9.25	0	1.75	3.26	5.34	358	27,475
Main variables								
Herding present								27,475
No	56.25							15,455
Yes	43.75							12,020
Number of funders investing since the campaign started	80.57	63.48	1	33	64	111	332	27,475
Average sum of investment during the previous 24 h	3175.65	5228.34	0	2000	2476.19	3222.22	282,333.3	27,475
Standard deviation of the sum of investments during the previous 24 h	3672.26	6984.86	0	1490.71	2499.75	3761.48	287,337	27,475
Funder investment (Danish Kroner)	3277.05	10,773.67	100	1000	1000	3000	781,000	27,475
Intensity (frequency of investments within the herd 24 h prior to investment)*	66.95	57.21	1	21	47	97	330	12,020
Persistency (duration of a specific herd*)	0.35	0.52	0	0.021	0.11	0.52	4.94	12,020
Log of Intensity*	3.79	0.98	0.69	3.09	3.87	4.58	5.80	12,020
Log of Persistency*	0.25	0.30	0	0.02	0.11	0.42	1.78	12,020

^+^The time of investment in a specific campaign varies based on the funder, not on the campaign level, and hence is funder-specific.

*The descriptive statistics show only values during “herding” periods.

### Herd operationalisation

To operationalize the herd itself, we draw on Li et al.’s^20^ iteration of the work of Lakonishok et al.^38^ on stock market trading and herding. Li et al.^20^ benchmark actual investments against a hypothetical scenario in which herd formation is absent. Assuming that the daily transactions for a campaign *i* in time *t* is *R*_*i,t*_, *R*_*i,t*_ should adhere to a Poisson distribution, where *T*_*i*_ is the fundraising duration of campaign *i*, if there is no herding and each investor makes decisions independently. According to Lakonishok et al.^38^, herding (LSV) is the difference between the observed deviation in daily investments and the scenario where herding does not exist, formulated as:

1 $$LSV_{i,t} = \left( {R_{i,t} - \rightthreetimes_{i} } \right) - E^{NH} \left[ { R_{i,t} - E\left[ {R_{i,t} } \right]} \right] = \left( {R_{i,t} - \rightthreetimes_{i} } \right) - 2{\text{exp}}\left( { - \rightthreetimes_{i} } \right)\frac{{\rightthreetimes_{i}^{{\left[ {\rightthreetimes_{i} } \right] + 1}} }}{{\left[ {\rightthreetimes_{i} } \right]!}}$$

where the first term is the observed deviation in the daily transaction amount for campaign *i*, and the second term is the expected value of the first term in the scenario where there is no herd formation. Consistent with Lakonishok et al.^38^, the initial herd for campaign *i* appears in time *t* when *LSVi*,*t* exceeds 0 for the first time, and disappears when *LSVi*,*t* falls below 0.

**Figure 3:**
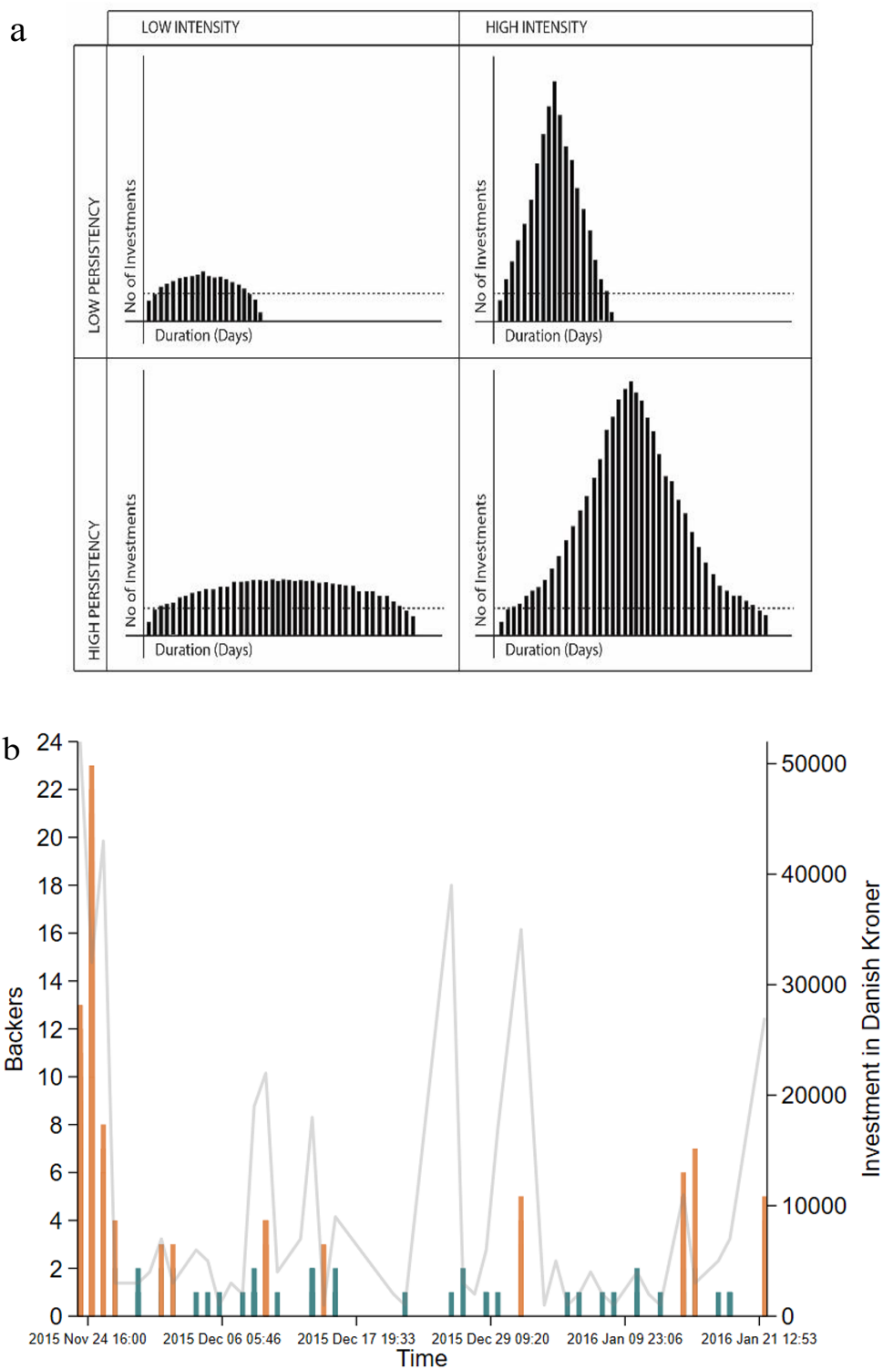
Macroscopic herd inputs: intensity and persistency. As visualized in panel (**a**), macroscopic herd inputs are measured in terms of their intensity and persistency, with intensity being the natural logarithm of the number of investors and persistency being the natural logarithm of the duration of the herd in days. These inputs will vary depending on the day on which an individual makes their investment. To gauge the effect of macroscopic herd input on individual investors as measured by the sums invested, we observe the intensity and persistency of the herd at the specific time on which an investor makes their investment. Panel (**b**) illustrates an exemplary crowdfunding campaign, with the y-axis on the left showing the number of investors (funders) by day, while total investment in DKK by day is shown on the right. The orange columns represent investments made during herding events, while blue columns represent non-herding investments, calculated according to the method developed by Lakonishok et al.^38^. The gaps in the graph represent days without investments.

To operationalize these macroscopic inputs as variables in our study, we draw on Sornette and Crane’s^39^ classification of collective human dynamics in distinguishing between herding as endogenous-critical or exogenous-critical. Endogenous-critical herding is characterized by significant precursory growth followed by almost symmetric relaxation, whereas exogenous-critical herding is marked by a sudden burst of activity followed by a rapid relaxation. A recent study by Li et al.^20^ conceptualized these herd dynamics in terms of “intensity” and “persistency”, with intensity denoting the frequency of investments within the herd at a specific point in time (in our case 24-h prior to investment) and persistency denoting the duration of a specific herd. See Fig. 3a for a visualization of these macroscopic herd inputs. Figure 3b illustrates an exemplary campaign denoting herding and non-herding investments.

### Inclusion and exclusion criteria

To increase the robustness of our model, we exclude outliers and implausible values in our data, i.e. we only focus on campaigns that that last for a maximum duration of 365 days (corresponding to 1.5 interquartile range), and we excluded investments during the first 2 min of the campaign to exclude automated investments. Table 1 provides a complete description of all variables in the final dataset used for the analysis (Table 2).

**Table 2:** OLS regression results for funder investments, depending on whether herding is present or not.

	(I)		(II)		(III)		(IV)		(V)	
	No Herding	Herding	No Herding	Herding	No Herding	Herding	No Herding	Herding	No Herding	Herding
Persistency during investment (logarithm of hours passed since herd started)	− 0.085***	0.070**	− 0.113***	0.052*	− 0.036**	0.026	0.017	0.099***	0.016	0.099***
	(0.013)	(0.030)	(0.014)	(0.031)	(0.017)	(0.032)	(0.016)	(0.035)	(0.016)	(0.035)
Intensity during investment (logarithm of the number of funders investing during the last 24 h)	− 0.186***	− 0.329***	− 0.139***	− 0.286***	− 0.073**	− 0.332***	− 0.049*	− 0.426***	− 0.046	− 0.407***
	(0.030)	(0.079)	(0.030)	(0.078)	(0.030)	(0.079)	(0.029)	(0.080)	(0.030)	(0.080)
Intensity during investment^2	0.017***	0.033***	0.013**	0.021**	0.008	0.038***	0.009*	0.062***	0.009*	0.060***
	(0.005)	(0.010)	(0.005)	(0.010)	(0.005)	(0.010)	(0.005)	(0.011)	(0.005)	(0.011)
Number of funders investing since the campaign started							− 0.002***	− 0.002***	− 0.002***	− 0.002***
							(0.000)	(0.000)	(0.000)	(0.000)
Average sum of investment during the previous 24 h									0.000*	0.000
									(0.000)	(0.000)
Standard deviation of the sum of investments during the previous 24 h									− 0.000*	− 0.000
									(0.000)	(0.000)
Campaign specific controls	No	Yes	Yes	Yes	Yes					
Funder specific controls	No	No	Yes	Yes	Yes					
R^2^	0.014	0.015	0.029	0.037	0.102	0.104	0.110	0.108	0.111	0.108
Observations	15,455	12,020	15,455	12,020	13,224	10,513	13,224	10,513	13,224	10,513

To account for potential variation in our results due to different durations in campaign length before they receive full funding and the number of funders financing a given project, we ran separate regressions to control for potential differences in our results. As can be observed in respectively Tables 3 and 4 the robustness of our results persisted across all models.

**Table 3:** Robustness check—OLS regression results for investor investments with varying minimum campaign lengths, given that herding is present.

	(I)	(II)	(II)
Campaign length	> 5 days	> 10 days	> 20 days
Persistency during investment	0.119***	0.129***	0.125***
	(0.039)	(0.043)	(0.047)
Intensity during investment	− 0.502***	− 0.436***	− 0.322***
	(0.098)	(0.101)	(0.100)
Intensity^2	0.073***	0.065***	0.049***
	(0.014)	(0.014)	(0.014)
Campaign specific controls	Yes	Yes	Yes
Investor specific controls	Yes	Yes	Yes
Number of funders since campaign started	− 0.001***	− 0.001***	− 0.001***
	(0.000)	(0.000)	(0.000)
Average sum of investment during the previous 24 h	0.000	0.000	− 0.000
	(0.000)	(0.000)	(0.000)
SD of the sum of investments during the previous 24 h	− 0.000	− 0.000	0.000
	(0.000)	(0.000)	(0.000)
Observations	6,277	5,223	4,205

Robust standard errors in parentheses. *p < 0.1, **p < 0.05, ***p < 0.01.

**Table 4:** Robustness check—OLS regression results for investor investments with varying minimum campaign sizes, given that herding is present.

	(I)	(II)	(III)
Campaign size (# funders)	> 70	> 105	> 118
Persistency during investment	0.104***	0.126***	0.128***
	(0.038)	(0.042)	(0.044)
Intensity during investment	− 0.192**	− 0.246**	− 0.275**
	(0.089)	(0.098)	(0.112)
Intensity^2	0.033***	0.041***	0.043***
	(0.012)	(0.013)	(0.015)
Campaign specific controls	Yes	Yes	Yes
Investor specific controls	Yes	Yes	Yes
Number of funders since campaign started	− 0.001***	− 0.001***	− 0.001***
	(0.000)	(0.000)	(0.000)
Average sum of investment during the previous 24 h	0.000	0.000	0.000
	(0.000)	(0.000)	(0.000)
SD of the sum of investments during the previous 24 h	− 0.000	− 0.000	− 0.000
	(0.000)	(0.000)	(0.000)
Observations	8788	6335	5603

Robust standard errors in parentheses. *p < 0.1, **p < 0.05, ***p < 0.01.

## Data Availability

The data can be provided by Lendino A/S pending scientific review and a completed NDA. Requests for the data should be submitted to: Kristian Roed Nielsen (krn.msc@cbs.dk). Data is anonymized employing unique ids.
